# Clinical Subtypes of Depression Are Associated with Specific Metabolic Parameters and Circadian Endocrine Profiles in Women: The Power Study

**DOI:** 10.1371/journal.pone.0028912

**Published:** 2012-01-03

**Authors:** Giovanni Cizza, Donna S. Ronsaville, Hayley Kleitz, Farideh Eskandari, Sejal Mistry, Sara Torvik, Nina Sonbolian, James C. Reynolds, Marc R. Blackman, Philip W. Gold, Pedro E. Martinez, for the P.O.W.E.R. (Premenopausal, Osteopenia/Osteoporosis, Women, Alendronate, Depression) Study Group

**Affiliations:** 1 Section on Neuroendocrinology of Obesity, National Institute of Diabetes and Digestive and Kidney Diseases, National Institutes of Health, Department of Health and Human Services, Bethesda, Maryland, United States of America; 2 Behavioral Endocrinology Branch, National Institute of Mental Health, National Institutes of Health, Department of Health and Human Services, Bethesda, Maryland, United States of America; 3 Nuclear Medicine Department, Warren G. Magnuson Clinical Center, National Institutes of Health, Department of Health and Human Services, Bethesda, Maryland, United States of America; 4 Research Service, Veterans Affairs Medical Center, Washington, D. C., United States of America; 5 National Institutes of Health, Department of Health and Human Services, Bethesda, Maryland, United States of America; University of Tor Vergata, Italy

## Abstract

**Background:**

Major depressive disorder (MDD) has been associated with adverse medical consequences, including cardiovascular disease and osteoporosis. Patients with MDD may be classified as having melancholic, atypical, or undifferentiated features. The goal of the present study was to assess whether these clinical subtypes of depression have different endocrine and metabolic features and consequently, varying medical outcomes.

**Methods:**

Premenopausal women, ages 21 to 45 years, with MDD (N = 89) and healthy controls (N = 44) were recruited for a prospective study of bone turnover. Women with MDD were classified as having melancholic (N = 51), atypical (N = 16), or undifferentiated (N = 22) features. Outcome measures included: metabolic parameters, body composition, bone mineral density (BMD), and 24 hourly sampling of plasma adrenocorticotropin (ACTH), cortisol, and leptin.

**Results:**

Compared with control subjects, women with undifferentiated and atypical features of MDD exhibited greater BMI, waist/hip ratio, and whole body and abdominal fat mass. Women with undifferentiated MDD characteristics also had higher lipid and fasting glucose levels in addition to a greater prevalence of low BMD at the femoral neck compared to controls. Elevated ACTH levels were demonstrated in women with atypical features of depression, whereas higher mean 24-hour leptin levels were observed in the melancholic subgroup.

**Conclusions:**

Pre-menopausal women with various features of MDD exhibit metabolic, endocrine, and BMD features that may be associated with different health consequences.

**Trial Registration:**

ClinicalTrials.gov NCT00006180

## Introduction

Major Depressive Disorder (MDD) is a common condition with a lifetime prevalence of 20.8% [1]. According to the Diagnostic and Statistical Manual of Mental Disorders, (DSM-IV), an episode of MDD can be classified clinically as depression with melancholic features and depression with atypical features. Melancholic depression is characterized by anhedonia, lack of reactivity to pleasurable stimuli and three or more of the following: loss of appetite or weight, insomnia, psychomotor retardation or agitation, sense of guilt, early awakening, depression that is worse in the morning, and a distinct quality of depressed mood. In contrast, atypical depression is defined by mood reactivity (mood brightens in response to positive events), appetite and weight increase, hypersomnia, leaden paralysis, and pathological sensitivity to perceived interpersonal rejection resulting in social or occupational impairment. To further define phenotypes of depressive episodes, distinctive features of clinical subtypes have been studied, including behavioral manifestations, response to antidepressants, regional differences in cerebral blood flow, perceptual asymmetries, electroencephalographic sleep recordings, and endocrine dysregulations [2]–[8]. More recently, cognitive impairment has been reported in subjects with melancholic depression [9]. MDD is characterized by a high level of clinical heterogeneity [10]; for this reason its construct as a nosological entity has been criticized [11]. The classification of episodes into clinical subtypes has been developed in an attempt to limit the clinical heterogeneity of MDD and is currently being re-evaluated in preparation of the DSM-V.

The epidemiology of clinical subtypes of depression has been characterized in the National Comorbidity Survey: 36% of individuals with MDD had atypical features of hypersomnia and hyperphagia, and 34% had melancholic features [12]. Atypical depression is more common in women [13], is associated with an earlier age of onset [2], [14], frequently includes comorbid anxiety [12], [14], or avoidant personality disorder [15], [16], and is associated with drug dependence, suicidal thoughts and attempts, utilization of health services, physical and sexual abuse or neglect, and functional disability [12]. In drug trials, the prevalence of atypical depression ranged from 16% to 42% [16]. Further, patients with atypical features respond better to monoamine oxidase inhibitors than to tricyclic antidepressants [4] and may have a smaller noradrenergic activation than patients with melancholic features [17].

It is becoming increasingly clear that MDD has a substantial impact on physical health [18]. In addition to osteoporosis [19], [20], cardiovascular disease [18], and immune alterations [21], MDD often co-exists with insulin resistance [18], diabetes [22] and obesity [23]. We conducted the POWER (Premenopausal, Osteoporosis, Women, Alendronate, Depression) Study, a longitudinal study of bone turnover in women with MDD, to determine whether major depression was associated with low bone mineral density. We have recently summarized the most important findings of this study [24]: MDD was associated with osteopenia, an increased risk for insulin resistance and cardiovascular disease, a chronic state of subclinical inflammation, increased diathesis to coagulopathy states, major endocrine, immune and neuropeptide alterations, and increased propensity to chronic pain.

The goal of this ancillary study was to determine whether different clinical subtypes of depression were associated with specific clinical or laboratory abnormalities. Thus, we characterized metabolic features, bone mineral density, and endocrine circadian profiles in the clinical subtypes of MDD using validated clinical markers. Our exploratory hypothesis was that individual clinical subtypes would be associated with different endocrine and metabolic features.

## Methods

### Participants

Study participants were premenopausal women, ages 21 to 45 years who enrolled in the POWER (Premenopausal, Osteoporosis, Women, Alendronate, Depression) Study, a longitudinal study of bone turnover in women with MDD [20]. Recruitment was conducted from July 2001 to February 2003 in the Washington, DC metropolitan area by advertising in newspapers, radio, Internet, and flyers. Written informed consent was obtained from all study participants. This study was approved by the Institutional Review Board of the National Institute of Mental Health.

### Psychiatric assessment

Participants were evaluated by clinicians trained in the use of the Structured Clinical Interview for DSM-IV for Axis I disorders, Patient Edition (SCID-IV I/P), version 2.0 [25], to diagnose unipolar MDD, according to criteria from the DSM-IV (American Psychiatric Association 1994) or to rule out any DSM-IV Axis I diagnosis (healthy controls). To minimize recall bias, an additional inclusion criterion for women with depression was having had an episode of MDD within the past three years. Melancholic and atypical subtypes were classified according to the most recent depressive episode. Patients that did not meet criteria for melancholic or atypical depression were classified as unspecified. Lifetime history of depression was estimated, including the number of depressive episodes and their length. Symptoms of depression and anxiety over the past week were assessed with the Hamilton Scale for Depression (HAM-D, 24 items) and the Hamilton Scale for Anxiety (HAM-A, 14 items).

Patients with bipolar disorder, current alcohol abuse or dependence, recent drug abuse or dependence, history of psychosis, anorexia or bulimia, or those at suicidal risk were excluded. Past or current comorbid anxiety and past binge eating disorder were not exclusionary criteria. As previously reported, 120 women with self-reported depression were screened, of whom 89 entered the study [20]. Women with MDD were classified according to the DSM-IV criteria as melancholic (n = 51) or atypical (n = 16). Depressed women whose last MDD episode had features of both melancholic and atypical depression, but did not meet criteria for either subgroup and those who met criteria for neither subgroup, were classified as having undifferentiated depression (n = 22). None of the patients with MDD had symptoms of psychosis, catatonia, or postpartum depressive features. Most patients were taking selective serotonin reuptake inhibitors (SSRIs), and no patient was taking tricyclic or monoamine oxidase inhibitors (MAOI). Sixty-three healthy controls were screened and of those, 44 women with no DSM-IV diagnoses other than past (not within the past five years) alcohol abuse were admitted into the study. Control subjects were excluded if they had any DSM-IV axis I diagnosis. Controls were matched to subjects with MDD based on age (±3 years) and BMI (±2 units), and blinded to the clinical subtype of subjects with depression. Except for two pairs, participants were matched by race as well.

### Medical assessment

All participants were in good physical health, as assessed by history and medical examination at screening. Menopause, defined as the absence of spontaneous menses during the preceding 6 months, was an exclusion criterion. Estrogen/progestin contraception was allowed, if unmodified for at least 6 months prior to enrollment.

### Anthropometric measurements

Body weight was measured to the nearest 0.1 kg using a platform digital scale; height was measured to the nearest 0.1 cm using a stadiometer. BMI was calculated as weight in kilograms divided by the square of height in meters (kg/m^2^) [26]. Waist circumference (WC) was measured with a non-stretch tape to the nearest 0.1 cm at the level of the uppermost lateral border of the right iliac crest. The mean of three measurements was used for analysis.

### Frequent blood sampling

Hourly blood collections were performed during a 24-hour period on 87 women who were available to complete an inpatient hospital stay: 32 with melancholic, 15 with undifferentiated, and 10 with atypical MDD features; and 30 healthy controls. Women were admitted to the inpatient unit the day before blood sampling began. Participants were placed on an *ad libitum* diet. Lights went off at 11 PM. Fasting blood was collected at 8 AM and then samples were taken at 1-hour intervals until 8 AM the next day for a total of 25 samples. Approximately one hour prior to sample collection, a catheter was inserted into a vein in the forearm, and the IV line was connected to the Venous/Arterial Blood Management Protection System (VAMP®, Edwards Lifescience Irvine, CA), a device that permits undiluted blood samples to be obtained from an in-line sampling site. The arm with the sampling catheter was maintained in a heating blanket to ensure arterialization of the venous sample. Blood samples were then placed in pre-chilled tubes using ethylenediaminetetraacetic acid as an anticoagulant. Approximately 0.3 liters of blood were drawn in total.

### Bone mineral density and body composition

Bone mineral density (BMD) and whole body composition were assessed by dual energy X-ray absorptiometry (DXA) scan, using the Hologic DXA QDR 4500 (Bedford, MA). The coefficient of variation for BMD was <0.4%. A radiologist (JCR) reviewed the DXA films and reported the percentage of fat for the whole body and the abdominal area extending from the T12/L1 interface to the L4/L5 interface, which includes both visceral and subcutaneous fat.

### Cooper test (12-minute walk/run test)

The Cooper test, which measures the distance covered during 12-minutes by running or walking as fast as possible, was used as an indirect index of physical fitness [26].

### Blood tests

Plasma ACTH and serum cortisol concentrations were measured using competitive chemiluminescent immunoassays (Nichols Advantage analyzer, San Juan Capistrano, CA, for ACTH; DPC Immulite-2000 analyzer, Los Angeles, CA, for cortisol) at the NIH Clinical Center Department of Laboratory Medicine. Inter-/intra-assay coefficients of variation (CV's) were all <10%. Plasma leptin was measured by ELISA (Linco Research Inc., St. Charles, MI) at Covance Laboratories (Vienna, VA), with inter-/intra-assay CV<10%. Fasting glucose and insulin levels were determined from the 8 AM sample, and insulin resistance was calculated using the homeostasis model for insulin resistance (HOMA-IR), a useful indicator of insulin resistance in patients with diabetes mellitus type 2 and an independent predictor of cardiovascular disease [27].

### Statistical analyses

Differences of means among groups were compared by analysis of variance (ANOVA), with pre-planned comparisons of each of the three MDD clinical subtypes with controls, using SAS PROC GLM (SAS Institute, Cary, NC). In case of a significant main group effect (control and the three MDD subtypes), post hoc comparisons between MDD subtypes were conducted using Tukey's least significant difference method. Categorical outcome measures were compared by Fisher's exact test.

The proportion of women with low BMD (t-score<−1.0) at the AP spine, femoral neck, or total femur was compared using logistic regression. To test for differences in the proportion of low BMD at any one or more of the three skeletal sites between women in each subtype and controls, a generalized estimating equation (GEE) model, in which the three bone sites are treated as repeated measures within each subject, was applied [28]. Analyses of BMD measures were adjusted for BMI.

Leptin and ACTH values were log transformed because of positive skewedness and were normalized, as verified by the Shapiro-Wilkes test. ACTH, cortisol, and leptin were each compared in repeated measures with mixed model analyses, using SAS PROC MIXED, where adjacent time-points were averaged for each subject, for a total of 12 values. In these analyses, the main effect of clinical subtype and its interaction with time of day were tested. Each depressive subtype was compared with controls in pre-planned contrasts. In case of a significant interaction, individual time points were compared and reported as significantly different if p<0.01. All data was reported as mean ± standard deviation (SD). Two-sided level of significance was set at a nominal 0.05 value, unless otherwise specified.

## Results

### Demographic characteristics and clinical features of depression

Table 1 reports the demographic and psychosocial characteristics. Women with undifferentiated MDD were slightly older and had greater BMI than controls. Women with atypical features were heavier, had an earlier age of menarche, were less often married, and less conditioned than controls. Women with melancholic features had demographic features similar to controls. Use of oral contraceptives, history of smoking, and alcohol use did not differ amongst the groups.

**Table 1:** Demographic characteristics of depressive subtypes of women with unipolar MDD.

		Depressive Subtype		
Characteristics	Healthy Controls (n = 44)	Undifferentiated (n = 22)	Atypical (n = 16)	Melancholic (n = 51)
Age (yrs)	34.7±6.8	38.2±5.4^b^	34.3±7.8	34.3±7.0
Body mass index (kg/cm^2^)	24.2±3.7	27.2±6.2^b^	28.9±7.1^a^	25.8±6.0
Weight (kg)	68.0±10.6	74.2±18.8	80.5±21.2^a^	70.4±17.3
White race	86.4%	90.9%	75.0%	90.2%
Years of education	16.3±2.1	16.9±2.0	15.1±1.9^a^	16.7±1.9
Married (%)	50.0%	54.6%	18.8%^a^	31.4%
Ever Smoked	31.8%	27.3%	50.0%	28.1% (9/32)
Age at Menarche	13.0±1.6 (n = 43)	12.7±1.6(n = 20)	11.9±1.6^a^(n = 15)	12.5±1.6 (n = 47)
Previous pregnancies	1.23±1.36	1.73±1.70	1.56±2.13	0.88±1.31
Contraceptive Pill Users	36.4%	18.2%	31.3%	35.3%
Estimated Total Calcium Intake (mg/day)	1408±755(n = 41)	1431±844(n = 19)	1571±699(n = 14)	1315±561(n = 45)
Alcohol Consumption (g/week)	6.9±11.6(n = 41)	5.0±4.8	3.9±6.1	4.3±6.2
Distance covered in the Cooper test (m)	1438±280(n = 23)	1252±253(n = 11)	1129±192^a^(n = 7)	1409±370(n = 20)

Values are reported as mean ± SD or percent.

Sample size in parenthesis, unless otherwise indicated.

Significant comparisons, assessed as p≤.05 nominal value.

**Overall**: overall test.

a Atypical differs from control.

b Undifferentiated differs from control.

The clinical characteristics are described in Table 2. Patients were, on average, mildly depressed or in clinical remission, as indicated by Hamilton depression and anxiety scores. However, about 17% of depressed women had experienced an episode of major depression within the past month (data not shown), and most had an average of four episodes and five years of cumulative time in depression. Age of onset of depression for the three subgroups was in the late teens. The most common comorbid Axis I diagnoses were anxiety disorders, present in 44% of depressed women and in similar proportions among the depressive subtypes. More than 80% of depressed patients were taking antidepressants, and approximately 15% used anxiolytics. Specifically, women with undifferentiated features had spent longer time in depressive episodes than women with atypical features.

**Table 2:** Clinical characteristics of depressive subtypes of women with unipolar MDD.

Clinical Characteristic	Undifferentiated (n = 22)	Atypical (n = 16)	Melancholic (n = 51)
Age of Onset	17.5±8.6 (n = 17)	21.6±9.6 (n = 14)	18.9±9.2 (n = 34)
Number of Depressive Episodes	4.5±2.3 (n = 17)	4.1±2.5 (n = 14)	4.4±2.8 (n = 34)
Months with Symptoms of Depression (Lifetime)	113.0±121.4 (n = 17)	35.7±33.8^b^ (n = 14)	59.8±54.4 (n = 34)
Global Assessment of Function (GAF)	65±9.1	61±8.2	62±10.0 (n = 49)
Hamilton Scale for Depression	9.7±8.0 (n = 19)	11.6±8.1	8.1±5.3 (n = 46)
Hamilton Scale for Anxiety	7.8±6.2 (n = 19)	7.7±4.8	6.5±4.2 (n = 46)
**Other Axis I Diagnoses**			
Post-Traumatic Stress Disorders	22.7%	12.5%	8.2% (n = 49)
Obsessive compulsive disorders	9.1%	6.3%	2.0% (n = 49)
Any anxiety disorder	50.0%	56.3%	36.7% (n = 49)
History of binge eating	4.6%	0%	2.0%
History of drug abuse	4.6%	37.5%	10.2% (n = 49)
History of alcohol abuse	31.3%	37.5%	16.3%
**Medications**			
Antidepressants	90.9%	81.3%	84.3%
Anxiolytic	13.6%	19.8%	11.8%

Values are reported as mean ± SD or percent.

Sample size in parenthesis, unless otherwise indicated.

Significant comparisons, assessed as p≤.05 nominal value.

b Atypical differed from undifferentiated.

### Metabolic, body composition, and bone mineral density measures

Women with undifferentiated features had more total fat, abdominal fat, and greater waist to hip ratio than controls (Table 3). Consistently, this group had a higher LDL, log triglycerides, and total cholesterol levels, as well as higher fasting glucose, insulin levels, and HOMA-IR compared to controls. After adjusting for whole body percent fat mass, the following remained significant: least squares adjusted means ± SD for undifferentiated vs. control, LDL, 123.6±27.0 vs. 108.0±27.1, p<0.05; log triglycerides, 4.7±0.50 *vs.* 4.3±0.51, p<0 .002; total cholesterol, 196.4±31.0 *vs.* 174.8±31.1, p<0.02; and fasting glucose, 93.5±9.5 *vs.* 88.1±9.5, p<0.05. Women with atypical features had more total body and abdominal fat, as well as greater waist/hip ratio than controls. Women with melancholic features did not differ from controls in their metabolic profiles.

**Table 3:** Metabolic, body composition, and bone mineral density measures.

		Depressive Subtype		
Characteristic	Healthy Controls (n = 44)	Undifferentiated (n = 22)	Atypical (n = 16)	Melancholic (n = 51)
**Body Composition**				
Whole Body Fat Mass (gm)	21887±6574	28155±11896^c^	30586±15164^a^	24716±10996
Whole Body Lean Mass (gm)	47228±6271	47409±8476	50712±8122	46303±7575
Whole Body % Fat	31.2%±5.5	36.1±6.5^c^	35.5%±9.3^a^	33.4%±7.7
Abdominal % Fat	22.7%±7.5(n = 30)	29.2%±9.6^c^(n = 15)	29.6%±12.5^a^(n = 11)	26.1%±10.1(n = 34)
Waist/Hip Ratio	0.75±0.05(n = 41)	0.81±0.05^c^(n = 19)	0.79±0.07^a^(n = 13)	0.77±0.05(n = 43)
Lipids	(n = 41)	(n = 19)	(n = 15)	(n = 46)
HDL (mg/dL)	58.8±11.5	55.9±15.5	55.9±14.7	57.3±12.8
LDL (mg/dL)	106.0±22.6	126.1±33.0^c^	118.8±31.9	116.2±28.4
Log Triglycerides (mg/dL)	4.25±0.40	4.76±0.69^c^	4.44±0.61	4.56±0.78^b^
Total Cholesterol (mg/dL)	173.4±25.7	198.1±35.8^c^	186.9±32.7	181.8±32.8
**Glucose metabolism**	(n = 41)	(n = 19)	(n = 15)	(n = 46)
Fasting Glucose (mg/dL)	87.6±9.5	94.1±11.8^c^	85.9±5.4	88.2±9.6
Insulin (mcU/mL)	6.06±4.28	9.72±8.16^c^	9.64±5.63	7.70±5.12
HOMA-IR	1.46±1.13	2.37±2.16^c^	2.03±1.11	1.72±1.29
**Percent prevalence of low BMD (T score<−1)**				
AP Spine	9.1	18.2	18.8	21.6
Femoral Neck	2.3	27.3^c^	18.8	11.8
Total Femur	2.3	18.2	12.5	13.7

Values are reported as mean ± SD or percent.

Sample size in parenthesis, unless otherwise indicated.

Significant comparisons, assessed as p≤.05 nominal value.

a Atypical differs from control.

b Melancholic differs from control.

c Undifferentiated differs from control.

Women with undifferentiated depression had a significantly higher prevalence of low BMD at the femoral neck than controls. Furthermore, GEE analysis indicated that low BMD at any of the three sites was more common in each subtype of depression than in controls. Odds ratios for low BMD were as follows: undifferentiated group: 6.81 (95% CI: 1.93–24.77, p<0.004); atypical group 5.83 (95% CI: 1.63–20.92, p<0.007); melancholic group: 4.22 (95% CI: 1.44–12.39, p<0.009). Higher mean 8 AM cortisol levels for all women were associated with low BMD (p<0.0001).

### Circadian ACTH, cortisol and leptin profiles

**Figure 1:**
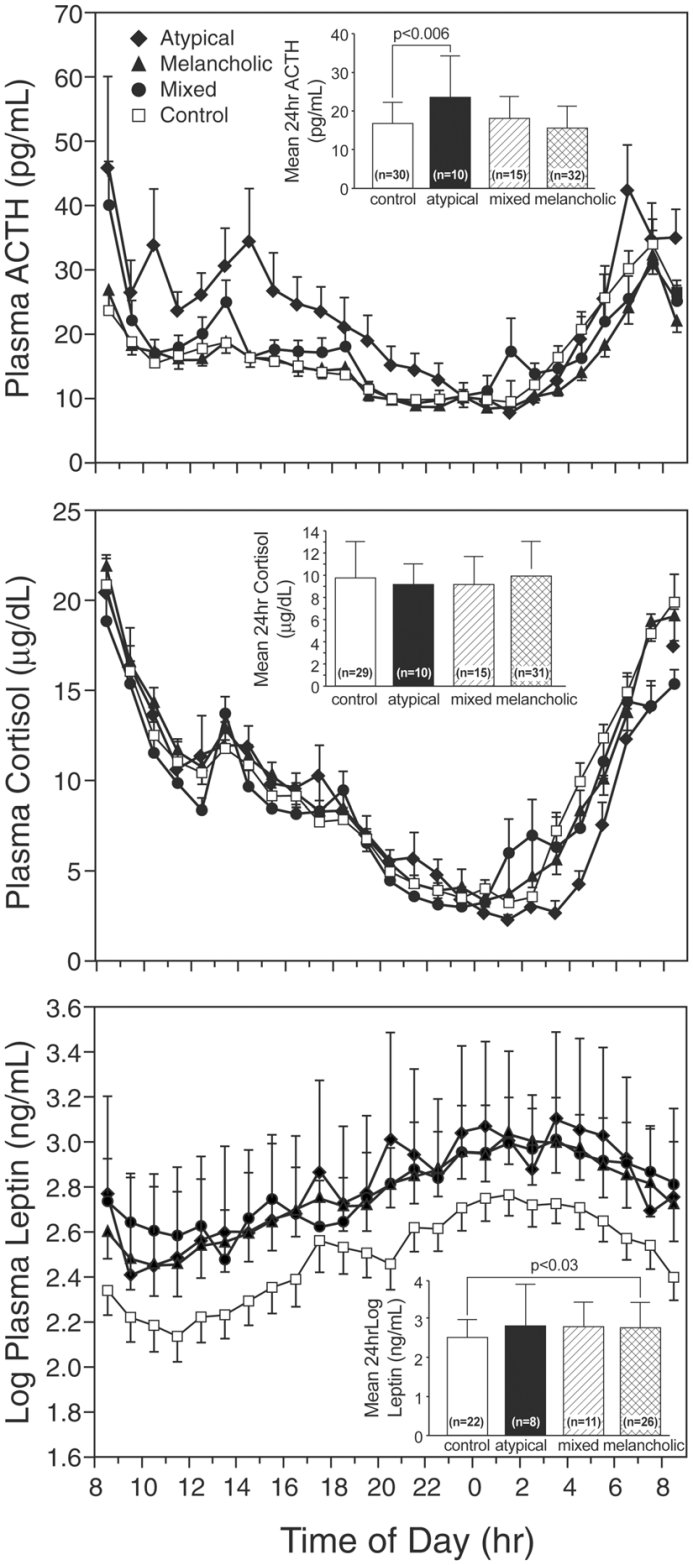
Circadian profiles of plasma ACTH, cortisol, and leptin.

Twenty-four hour plasma ACTH, cortisol, and leptin secretion are depicted in Figure 1. Mean 24-hour plasma ACTH concentrations were higher in the atypical subtype vs. the control group (F (1, 83) = 4.01, p<0.05), as well as in the melancholic vs. control group (F (1, 83) = 6.89, p = 0.01). In addition, a significant group by time interaction (*F* (33, 909) = 2.38, *p*<0.0001) revealed that ACTH was elevated in the atypical group only in the daytime (Figure 1), with the greatest differences between atypical and controls from 10 AM to 5 PM (all p<0.01). These differences remained significant after adjustment for BMI.

No differences in mean 24-hour plasma cortisol values were observed. After adjustment for total body fat, the mean 24-hour adjusted log leptin value was elevated in the melancholic subgroup, as compared with controls (adjusted means = 2.84 melancholic vs. 2.63 control; F (1, 66) = 5.17, p<0.03).

## Discussion

The three different phenotypes of depression exhibited distinct features compared to control subjects; women with atypical or undifferentiated MDD exhibited greater BMI, waist/hip ratio, whole body and abdominal fat mass, and higher mean 24-hour ACTH levels. (Table 4). Women with melancholic features had higher mean 24-hour leptin levels than controls. Finally, women with undifferentiated depression exhibited higher fasting lipids, glucose, and insulin levels; and had a greater prevalence of low BMD at the femoral neck than controls.

**Table 4:** Synopsis of significant findings in the three subtypes of depression vs. healthy controls.

	Undifferentiated	Atypical	Melancholic
BMI		⇑	
Weight		⇑	
Percent married		⇓	
Age at menarche		⇓	
Physical conditioning (Cooper Test)		⇓	
Months of symptoms of depression (Lifetime depression)		⇓	
Waist circumference		⇑	
Fat mass	⇑	⇑	
Abdominal fat mass		⇑	
Fasting glucose	⇑		
Fasting insulin	⇑		
HOMA	⇑		
LDL	⇑		
Triglycerides	⇑		⇑
Total cholesterol	⇑		
Prevalence of low BMD	⇑ (femoral neck)		
ACTH (After adjustment for BMI)		⇑	⇑
Leptin (after adjustment for body fat)			⇑

### Metabolic features

Both women with atypical or undifferentiated depression were heavier than controls. The greater BMI, waist circumference, and fat mass are consistent with the hyperphagia that distinguishes atypical depression. The NESDA Study, a large cohort study conducted in the Netherlands reported also higher body weight, a greater prevalence of metabolic syndrome, and more somatic symptoms in subjects with atypical depression [29]. Women with undifferentiated depression had significantly higher fasting glucose and insulin and HOMA index [30]. Specifically, the average HOMA index in this subgroup, 2.4, approached the accepted cut-off for insulin resistance, 2.6. In keeping with our findings, a recent meta-analysis reported a strong association between depression and type 2 diabetes, with a 60% increased risk in depressed subjects of developing diabetes over time [31]. We also examined lipid parameters: subjects with undifferentiated depression had higher LDL, tryglicerides and total cholesterol than controls. Some of the altered measures are part of the definition of metabolic syndrome;. MDD and metabolic syndrome co-occur, and may be related to each other in a bi-directional way, as they share common pathogenetic mechanisms, including elevated proinflammatory cytokines and glucocorticoids [32]. Whether women with undifferentiated depression have a greater risk for diabetes and cardiovascular disease than other clinical subtypes of depression, particularly after menopause when losing the protective effects of estrogens, remains to be determined in prospective studies.

### Endocrine features

#### HPA Axis

We measured hourly plasma levels of ACTH and cortisol for 24 hours in different subtypes of depression. Alterations of the HPA axis in depression are a reliable finding in biological psychiatry. These abnormalities are however mostly observed during the depressive episodes and relate to clinical severity. The typical circadian profile of ACTH and cortisol, two pulsatile hormones, was preserved in the three clinical subtypes. This is consistent with the fact that most of these women were not depressed at the time of sampling and in clinical remission. We nevertheless observed some subtle differences compared to controls. Women with melancholic and atypical features of depression had higher plasma ACTH levels compared with controls. Higher plasma ACTH may be secondary to a dysregulation of the CRH hypothalamic neuron [33], reduced negative feedback of the pituitary corticotroph by cortisol, impaired response of the adrenals to ACTH, as well as a combination of all these factors. Although the majority of these patients were taking antidepressants, it is unlikely that higher plasma ACTH levels were due to pharmacological treatment as higher plasma ACTH has been reported in a cohort of women with untreated atypical features of depression [34]. A recent meta-analysis found no differences in ACTH levels in clinical subtypes [35]; however repeated sampling over 24 hours may unravel abnormalities not evident with single plasma sampling. Alterations in ACTH secretion have been reported in depression; with 10 min sampling over 24 hours Young et al. [36] demonstrated less “orderly” ACTH secretion, as indicated by increased entropy in non-medicated premenopausal women with depression.

#### Plasma leptin

Women with melancholic depression had higher leptin levels compared to controls. In a related study, we reported that 24-hour plasma leptin levels were about 30% higher in a subset of 23 women with depression compared to 23 BMI -matched controls [37]. Because leptin provides satiety cues to the brain [38], it may explain the lack of appetite in melancholic depression. Higher levels of plasma leptin in patients with melancholic depression may have been caused by increased sympathetic tone. Catecholamines stimulate leptin secretion [39] and CSF and plasma norepinephrine and epinephrine are increased in patients with melancholic depression [40]. A leptin hypothesis linking mood disorder and obesity, two largely overlapping conditions at the population level, has been formulated [41]. Extra-hypothalamic actions of leptin are beginning to emerge; these include antidepressant and mnemonic effects [42]. Inappropriately higher leptin levels for body weight in depressed subjects are reminiscent of inappropriately elevated leptin levels in obese subjects. Future studies should determine if subjects with depression develop a resistance to the antidepressant effects of leptin when gaining weight.

#### Reproductive history

Women with atypical depression had menarche approximately one year earlier (12 vs. 13) than control subjects. This age was, however, well within normative age, which is 12.5 in the US, based on the NHANES data [43]. In addition, given the study design, sampling bias may have contributed to the difference in age at menarche. Nevertheless, it should be noted that the risk of depression increases with decreasing age at menarche, as data from the Harvard Study of moods and cycle, a large population-based study, suggested using a nested case-control approach [44].

### BMD features

The existence of an association between major depression and osteoporosis has been well documented [19], [45], and the endocrine and immune mechanisms at play have been summarized in recent reviews [24], [46]. In this study we analyzed the relationship between major depression and osteoporosis based on clinical subtypes. We found that osteopenia at the femoral neck was more prevalent in women with undifferentiated features than in controls. Higher morning cortisol levels were associated with low bone mass, but did not discriminate between the subtypes.

### Study limitations and strengths

The following study limitations should be noted. Most women were on antidepressants, anxiolytics, and/or oral contraceptives; these medications may have affected some of the outcome measures. In addition, the frequency of blood sampling did not allow for hormone pulsatility analyses, and no dynamic endocrine testing was performed. Since the study was not specifically designed to assess phenotypic differences in clinical subtypes, the sample size was uneven among groups. The current analysis of phenotypic differences among clinical subtypes was of exploratory nature and the current findings should be regarded as hypothesis generating. On the other hand, this sample was prospectively assembled; homogeneous; carefully characterized in terms of clinical, endocrine, metabolic, and bone mass parameters; and a relatively large number of subjects underwent frequent sampling.

### Overall clinical and pathophysiological relevance

The current findings suggest that among the three clinical subtypes, undifferentiated depression may carry the greatest cardiovascular morbidity and increased risk of diabetes and hip fractures. Historically, the construct of depressive subtypes was developed based on clinical symptoms and response to antidepressants. This study suggests that these subtypes may indeed be biologically rooted and have distinct medical consequences.

In summary, we found that different clinical subtypes of depression were associated with specific bone, metabolic, and endocrine features. Our findings lend support to the idea that depressive subtypes are not mere clinical entities but are rather distinct nosological entities, possibly underlying a differential dysregulation of the CRH hypothalamic neuron in depressive subtypes. Future studies should determine the extent to which these distinct features translate into differential medical morbidity, the genetic predisposition to different clinical phenotypes, and the stability of these traits over the lifetime.
